# Parent/caregiver needs during pediatric genome‐wide sequencing: A scoping literature review

**DOI:** 10.1002/jgc4.70209

**Published:** 2026-04-30

**Authors:** Priyanka Murali, Joon‐Ho Yu

**Affiliations:** ^1^ Division of Genetic Medicine, Department of Pediatrics University of Washington Seattle Washington USA; ^2^ Institute for Public Health Genetics Seattle Washington USA; ^3^ Seattle Children's Hospital and Research Institute Seattle Washington USA

**Keywords:** caregivers, exome sequencing, genome sequencing, parents, pediatric genetics

## Abstract

The integration of genome‐wide sequencing (GWS) including whole‐exome and whole‐genome sequencing, has transformed pediatric diagnostics, yet the needs of parents and caregivers during this process remain insufficiently explored. This scoping review aims to synthesize current knowledge on parental and caregiver needs across the GWS process in pediatric settings to inform better clinical practices and support systems. A scoping review was conducted following PRISMA guidelines. Electronic databases including PubMed, PsycINFO, CINAHL, Embase, and Web of Science were searched, yielding 684 articles, with 57 meeting the inclusion criteria. Data extraction focused on study characteristics, clinical settings, and identified parental needs categorized into pre‐test, interim, and post‐test periods. Conventional content analysis was used to inductively code and identify categories of needs. Parental needs were categorized into two main themes: (1) informational needs, encompassing tailored communication, understanding prognosis, logistics, and evolving information; and (2) emotional support, emphasizing the importance of initial provider interactions and support from healthcare providers and peer groups. Informational and emotional needs were interrelated, impacting parents' overall experiences. The review highlighted significant gaps during the interim waiting period, with needs largely focused on pre‐ and post‐test periods. Parents navigating the pediatric GWS process require comprehensive informational and emotional support. Effective communication before testing and empathetic follow‐up contribute to positive experiences. Addressing gaps at different times throughout the process and fostering continuous provider and peer support can enhance the integration of GWS in pediatric care, improving family‐centered outcomes.

**What is known about this topic**

Parents and families going through the process of genetic testing have different support needs that arise, related to both medical and non‐medical systems. Much of the literature so far has focused on genetic testing broadly and different parental perspectives and experiences as they go through these processes.

**What this paper adds to the topic**

This paper identifies and synthesizes different support needs as parents and families undergo the process of genome‐wide sequencing (exome and genome). It examines not only different categories of needs but also needs at different points of time in the testing process.

## 1 INTRODUCTION

Advances in genetic and genomic testing have transformed the landscape of disease diagnosis, treatment, and prevention (Horton & Lucassen, 2019). The development of these technologies has generated new opportunities for improved diagnosis of genetic disorders as well as targeted treatments in various clinical contexts (Naidoo et al., 2011). Technologies like whole‐exome and whole‐genome sequencing (together termed genome‐wide sequencing or GWS) are being used for pediatric patients with heterogeneous medical presentations, given higher rates of diagnostic yield compared to previously available molecular and cytogenetic testing (Moore & Richer, 2022). However, there are barriers that prevent parents and caregivers of pediatric patients from easily navigating medical and non‐medical systems as they go through the process of genetic testing (Dusic et al., 2022), related to a lack of support at different points along the genetic testing process, including differential access, challenges to obtaining informed consent and returning results, as well as gaps in healthcare infrastructure (Boothe et al., 2021; Maiese et al., 2019; Nisselle et al., 2021). As such, the clinical translation of this research requires a deeper understanding of how the implementation of genetic testing technologies is confronted by systemic problems that challenge the adoption of new technologies by patients and their families.

GWS testing is often delineated into pre‐ and post‐test periods, which can require different forms of counseling (Elliott, 2020; McGlynn & Langfelder‐Schwind, 2020). The pre‐test period begins with an explanation of the method of testing used, the associated risks and benefits, and a discussion about the range of results that can be generated (McGlynn & Langfelder‐Schwind, 2020). In the interim period, a sample is collected while individuals wait for results. In the posttest period, or the time following return of results, information is shared, and the families' understanding is assessed and expanded, potentially creating a medical management and treatment plan (McGlynn & Langfelder‐Schwind, 2020). The needs of parents of pediatric patients change as they go through this process, given that both the information and the context in which they receive it differ in these periods (Rink & Kuller, 2018).

Studies in the context of single gene testing have identified mitigation of anxiety and a need to better understand current and future impact of genetic testing results as parental needs during this process (Grosfeld et al., 2000; Hendriks et al., 2005). However, to the best of our knowledge, no review of the current literature has examined the needs of parents and caregivers of pediatric patients as they have navigated GWS testing across different clinical contexts. Additionally, parental needs and their similarities or differences across different time periods of the GWS process have yet to be explored. Synthesizing this information may be useful in understanding how to improve parent experiences of GWS and at what time points during the GWS process to target potential interventions. We contend that as GWS becomes increasingly integrated into medical care, failing to understand patient‐stakeholder perspectives may hinder effective implementation and exacerbate existing barriers to appropriate genetic and associated follow‐up care.

Therefore, we conducted a scoping review of the peer‐reviewed academic literature to query the support needs of parents and caregivers of pediatric patients undergoing GWS. The primary aim of this review was to synthesize existing research throughout the GWS process. Additionally, we aimed to identify patterns or variations in these needs at different stages of the GWS journey. By doing so, we hope to inform future research and clinical practices that can more effectively address the unique challenges faced by families navigating pediatric GWS.

## 2 METHODS

### 2.1 Scoping review

We undertook a scoping review of the literature. A scoping review examines the extent, range, and nature of a particular activity and works to identify gaps in the existing literature (Levac et al., 2010). Scoping reviews are useful for examining emerging evidence when it is still unclear what more specific questions can be posed and addressed by a systematic literature review. Given that the state of support needs in this specific context has not fully been examined, a scoping review will be useful to determine the depth and breadth of knowledge regarding this particular topic. This work was conducted as a part of a doctoral dissertation, not as an independently funded research project. Findings were reported in accordance with the Preferred Reporting Items for Systematic Reviews and Meta‐Analyses extension for Scoping Reviews (PRISMA‐ScR) (Appendix S4).

### 2.2 Scoping review questions

We asked the following question: “What is the current state of knowledge about the needs of parents and caregivers throughout the process of GWS in pediatric patients?”

Additionally, a protocol was developed by the first and second authors with assistance from a research librarian (see Appendix S1). It was not prospectively registered for this scoping review. Protocol registration for scoping reviews was not yet standard practice or required by most journals at the time this project was being developed in late 2022/early 2023.

### 2.3 Search strategies

With the assistance of an academic research librarian, an initial search of electronic databases and gray literature was conducted. We generated search terms, built search strings, and conducted searches tailored to specific databases. PubMed, PsycINFO, CINAHL, Embase, and Web of Science databases were searched. Search terms were generated by first identifying concepts that encompass different parts of the research question. These concepts included: “pediatrics,” “whole genome sequencing,” “parental or caregiver,” and “attitudes/needs/perspectives.” Relevant MeSH terms were identified based on these concepts of interest. Synonyms to MeSH terms were brainstormed and searched within each database. Some search strings were pre‐built for specific databases. Other search strings were built by the first author and the research librarian. Terms for other databases were modified to meet the specifications of the particular database. When terms from one database did not have an equivalent in other databases, those terms were included as free text in the search string. Full search strings for each database are included in the Appendix S3. Titles and abstracts of all articles were searched.

### 2.4 Inclusion and exclusion criteria

#### 2.4.1 Population

Parents and caregivers of pediatric patients (aged 0–18 years) who had undergone GWS, defined as either whole exome or whole genome sequencing, were included. Articles with multiple participant groups (e.g., pediatric patients, clinicians, or lay people in addition to parents/caregivers) were included if the data for each group could be isolated (Appendix S2).

#### 2.4.2 Concept

This review examined parent and caregiver experiences, expectations, perceptions, and needs related to GWS. Needs were stratified based on timing: preceding sample collection, the interim between sample collection and return of results, or following return of GWS results. Stakeholder interactions were also examined. Articles were required to have returned GWS results to parents/caregivers for inclusion.

#### 2.4.3 Context

Studies conducted across various clinical subspecialties and geographic locations were included, with no restrictions on setting.

#### 2.4.4 Types of sources

English‐language articles employing qualitative, quantitative, or mixed methods study designs were included.

This information is included in table format and can be found in the Supplemental materials.

### 2.5 Study selection

Searches were conducted on May 2, 2023, by the first author in the following databases: PubMed, PsycINFO, CINAHL, Embase, and Web of Science. Search results were imported into the Zotero reference management platform. Duplicates were removed prior to uploading the titles and abstracts into Covidence, an online literature review management system. Titles and the abstracts of uploaded articles were screened for inclusion criteria; if met, a full text review was conducted by the first author. The second author was consistently kept abreast of how many and what type of articles were being included in the screening process. There were frequent, periodic check‐ins between the first and second author, and the second author reviewed the screening process to determine if there was any disagreement in the way articles were being screened.

### 2.6 Data extraction

The following data were extracted from each included study using an extraction template: (a) bibliographic details (author, year, title, journal, DOI), (b) geographic location, (c) study aim, (d) study design, (e) clinical subspecialty in which study was conducted, (f) participant relationship to child, (g) total number of participants in the study, (h) parent/caregiver experiences, expectations, and perceptions, (i) needs preceding, during, and following GWS (if explicitly stated), and (j) stakeholder interactions. The first and second authors jointly reviewed the data extraction template and collaboratively extracted data from initial articles to establish a consistent process. Following this phase, the first author completed the remaining extractions with frequent check‐ins with the second author, who periodically reviewed extracted data to ensure accuracy and consistency.

The extracted outcomes of this scoping review are parent/caregiver experiences, expectations, and perceptions, and parent/caregiver needs at different points during the process of whole genome sequencing. Data about needs throughout the process of GWS testing were stratified based on whether this was a need preceding sample collection and GWS of the sample, during (in the interim between sample collection and return of results), or following return of GWS results. Extracted data were exported from Covidence into an Excel worksheet.

### 2.7 Data synthesis and analysis

Each article was reviewed three times to determine if all the data had been extracted and all relevant concepts captured. Excel was then used to calculate frequencies of different categories of extracted data.

### 2.8 Data analysis

We conducted a conventional content analysis (Hsieh & Shannon, 2005) of identified primary research articles to describe the state of knowledge of parents' needs throughout the process of pediatric GWS. As is typical with this approach, codes were developed inductively and sorted into categories based on how they were related and linked.

The extracted data from the needs preceding, during, and following GWS categories were imported into Atlas.ti. 23.2.1 for Mac. Initial codes were developed based on a review of the data extracted from the first 10% of articles. The data were then coded line by line, and more codes were added as necessary based on the content of the data. The data were then reviewed again to ensure that all relevant concepts had been captured. These codes were then sorted into appropriate descriptive categories and ultimately, broader thematic categories. Specifically, the analysis focused on how the codes applied across all need categories were similar or different. These categories were further refined and finalized following discussion between the first and second authors.

## 3 RESULTS

### 3.1 Search outcome

**FIGURE 1:**
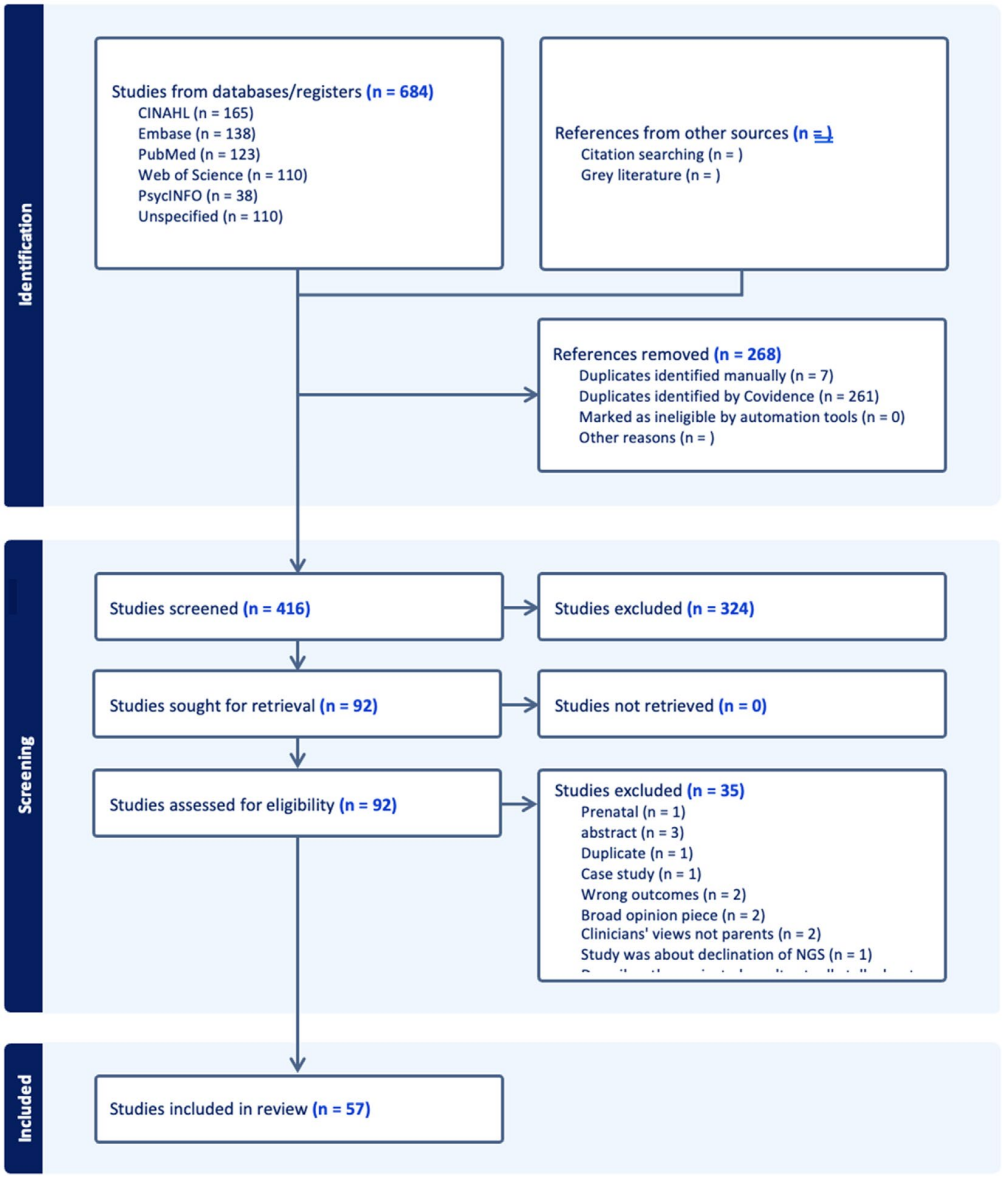
Flow diagram following PRISMA guidelines demonstrating the results of the literature search.

The initial database search returned 574 records. Once duplicates were removed, there were 338 articles remaining for title and abstract screening. Of those, 270 were deemed irrelevant based on the inclusion and exclusion criteria. This left 68 articles to be assessed for full‐text eligibility. A total of 47 articles reporting findings from 47 independent studies were eligible for inclusion in the final scoping review. An updated search was conducted on December 3, 2025, to capture any potentially relevant studies that were published since the initial search. This added 10 articles that met the inclusion criteria and for which full extraction was completed, bringing the total number of articles in the review to 57. Figure 1 provides a flow diagram following the Preferred Reporting Items for Systematic Reviews and Meta‐Analyses (PRISMA) guidelines of the search and screening process, including articles from the updated search.

### 3.2 Article characteristics

The included articles provided data on the needs, experiences, expectations, and perspectives of parents/caregivers of pediatric patients who underwent GWS. These articles included studies that were conducted across six countries: the United States, Canada, the United Kingdom, the Netherlands, Australia, and Japan. Eight articles focused on GWS in NICU/neonatal settings, seven in oncological settings, six in rare disease settings, four in developmental settings, and four in neurological settings. Four articles involved GWS across multiple clinical settings, and the remaining seven articles addressed various other medical settings, such as cardiac anomalies, congenital malformations, and newborn screening.

Twenty‐eight articles employed qualitative methods, 16 utilized mixed methods, six used surveys, and the remainder used other approaches, including but not limited to focus groups, review of EMR, and clinical observation. Table 1 provides this information, while Table 2 provides a summary of included articles.

**TABLE 1:** Article characteristics.

	*N* (%)
Country study was conducted	
Australia	3 (5.3)
Canada	11 (19.3)
United Kingdom	7 (12.3)
United States	30 (52.6)
Netherlands	5 (8.8)
Japan	1 (1.8)
Total	57
Clinical settings of studies	
Developmental	4 (7)
Multiple settings	4 (7)
Neurological	5 (8.8)
Neonatal/NICU	10 (17.5)
Oncological	7 (12.3)
Other	19 (33.3)
Rare disease	8 (14)
Total	57
Study design	
Interview—qualitative	28 (49.1)
Mixed methods	16 (28.1)
Survey	6 (10.5)
Other	7 (12.8)
Total	57

**TABLE 2:** Summary table of articles.

Author, year	Country	Study aim	Study design	Study sample	Experiences/needs
Alam et al. (2022)	Canada	To examine the experiences of parents of children with early‐onset epilepsy who pursued whole‐exome sequencing (WES) and to determine the impact of test results on their child's treatment	Qualitative (interview)	25 parents 21 children	Parents wanted to better understand the prognosis, a big motivator to pursue WES. They desired peer support and clarify regarding secondary findings
Aldridge et al. (2021)	Canada	To understand whether parental perceptions of genom‐wide sequencing (GWS) utility differ based on the diagnostic and living/deceased status of the child	Qualitative (interview)	14 parents	Parents appreciated getting a diagnosis, given that it alleviated guilt and also informed a care plan for their child. They also desired peer support
Anderson et al. (2017)	Canada	To understand ethical considerations related to GWS in a pediatric setting	Qualitative (interview)	25 parents	Parents valued the GWS journey to provide more information on their child's diagnosis. They wanted more clarity regarding IFs
Armstrong et al. (2022)	United States	To determine parental attitudes regarding population‐based newborn screening (NBS) and newborn genomic sequencing	Quantitative (survey)	248 parents of 174 newborns	Parents are supportive of NBS and feel that thorough informed consent is critical
Berrios et al. (2020)	United States	To understand parental responses to the use of GWS for infants with suspected genetic disorders	Mixed methods	23 parents completed the survey 22 parents completed the interview	Parents feel overall positive about GWS for their children
Biesecker et al. (2025)	United States	To understand and characterize the perspectives of parents from historically underserved backgrounds regarding the implementation of genomic medicine	Quantitative (survey)	1763 parents	Parents reported lower understanding when results were uncertain; perceived utility did not differ significantly depending on whether or not the first language was English, although those from medically underserved backgrounds felt greater overall perceived utility
Bon et al. (2022)	The Netherlands	To explore families' motives, knowledge, and views regarding germline genetic sequencing to improve future counseling and support	Qualitative (interview)	29 parents	Parents are very motivated to pursue genetic testing for their child and perceive minimal burden regarding sequencing. Some have anxiety about results, but still feel positively
Brett et al. (2019)	Australia	To explore parental experiences of ultrarapid genomic testing for their critically unwell children	Quantitative (survey)	55 parents	Parents desire tailoring pre‐ and post‐test counseling to their mental state. Some reported a lack of adequate info in the post‐test period
Cakici et al. (2020)	United States	To understand parents' perceived utility, adequacy of consent, and potential harms/benefits of rapid diagnostic genome sequencing for infants in the NICU	Quantitative (survey)	312 parents from 213 families	Parents reported adequate info at the time of informed consent and little decision regret following. They reported being well‐informed about testing procedures and their child's health
Cheung et al. (2022)	Canada	To explore the experience of parents who chose to receive secondary findings from GWS and the long‐term impact on their families	Qualitative (interview)	12 parents	Much of the focus was on pretest counseling and how secondary findings were introduced
Childerhose et al. (2021)	United States	To speak with parents about the experience of a diagnostic odyssey for their children with intellectual disability or developmental delay	Qualitative (interview)	59 parents	Parents seek therapies and services beyond the clinic; Parents require a diagnosis to obtain school services and optimize placements; Parents leverage a genetic diagnosis in their quest for therapies and services; A specific molecular diagnosis is more useful than a general one like IDD
Cornelis et al. (2016)	The Netherlands	To understand parents' preferences for unsolicited findings (UF) from diagnostic WES for their children	Qualitative (interview)	34 parents	Parental discussion centered around UF specifically and not WES broadly. Considerations favoring the disclosure of UFs included: availability of medical treatment and prevention, utility beyond medical intervention
Cornelis et al. (2016)	The Netherlands	To compare parents' attitudes regarding exome sequencing (ES) before and after results disclosure	Qualitative (interview)	16 parents	Parents stated that receiving a diagnosis brought relief along with sadness. Having a label often opened doors to different types of support
Dolling et al. (2025)	United Kingdom	To evaluate parents' psychosocial needs and to explore their support experiences	Missed methods	63 families/91 parents	Parents of medically complex children experienced significant vulnerability and barriers to support, with those facing the greatest complexity reporting the least. They desired holistic support from healthcare providers that addressed both medical and parental well‐being, with peer support viewed as supplementary rather than primary
Gurasashvili et al. (2024)	United Kingdom	To explore the parental experience of receiving a “no primary finding” (NPF) result following genome sequencing (GS)	Qualitative (interview)	12 parents	Families described a consistent pursuit of a diagnosis with increasing feelings of hope as they navigated their GS journey
Halley et al. (2022)	United States	To characterize the range of benefits and potential harms of GS from the perspectives of families with diverse sociodemographic characteristics	Qualitative (interview)	30 parents	Parents of diagnosed and undiagnosed children did perceive GS as having utility in terms of positive impacts on parents' psychological well‐being, on their child's healthcare management, and through facilitating disease‐specific social connections and research opportunities
Hill et al. (2020)	United Kingdom	To explore parent and professional perspectives around the usefulness of rapid genome sequencing (RGS), as well as the potential for unintended harms and challenges	Qualitative (interview)	11 parents 19 professionals	Parents saw positive and negative clinical and psychosocial benefits to RGS in many forms
Hitchcock et al. (2020)	Canada	To understand stakeholder perspectives on the content, use, and length of consent forms for GWS in a clinical research setting	Qualitative (focus group)	8 parents 7 healthcare providers	Parents wanted consent forms to focus on immediate and future (post‐return of results or RoR) implications of GWS
Inglese et al. (2019) (CAUSES)	Canada	To explore the family experience related to having a child diagnosed with a newly discovered syndrome (via WES)	Qualitative (interview)	12 parents	Parents of children undergoing WES felt like there was a lack of information as well as a need for resources and support (at school, respite care). Lack of social support could lead to feelings of isolation
Krabbenborg, Schieving, et al. (2016)	The Netherlands	To explore the parental factors for ES of children with neurological conditions	Qualitative (interview)	50 parents	Parents desired clarity in understanding the ES process, from informed consent to post‐RoR
Krabbenborg, Vissers, et al. (2016)	The Netherlands	To understand the experience of positive, negative, or inconclusive ES results in the pediatric rare disease context	Qualitative (interview)	26 parents	Parents suggested that personal utility depended on clinical context and timing of testing
Lee et al. (2022)	Canada	To explore the personal utility (defined by Kohler et al., 2017) of GWS for parents of children with medical complexity (CMC)	Qualitative (interview)	15 parents	Parental expectations did not differ among those whose children were diagnosed or not diagnosed. Parents expected utility in emotional, information, and knowledge‐based ways. Expectations of GS were related to previous genetic testing and how healthcare providers introduced the concept of GS
Lemke et al. (2023)	United States	To understand how the outcomes involving utilities and disutilities might differ between the NICU and sequencing performed later in childhood, and how a molecular diagnosis fits into the broader story for families	Qualitative (interview)	62 families/78 individuals	Parents discussed that receiving genomic results in infancy helped families prepare for future care and provided answers, though it also affected parent–child bonding and resulted in guilt, underscoring the importance of psychosocial support and thoughtful pretest counseling communication
Lewis et al. (2020)	United Kingdom	To explore attitudes of parents participating in the 100,000 Genomes Project (a project in which participants are offered genome sequencing for cancer and rare and inherited disease diagnosis) towards GS	Qualitative (interview)	20 parents	Parents wanted more understanding regarding the use of personal genomic data protections and privacy during the consent process, and more time to discuss and understand the implications of secondary findings
Li et al. (2019)	United States	To explore the psychosocial impact of receiving a VUS from pediatric ES and to identify implications for clinical practice	Quantitative (survey)	14 parents	Parents emphasized the importance of accessibility of the clinical and needing a personal touch from the clinical care team
Liang et al. (2022)	United Kingdom	To quantify post‐test decisional regret and characterize long‐term post‐test experiences and unmet needs of the parents of children with suspected genetic diseases after they had received GWS results	Mixed methods	32 parents	Parents reported little decisional conflict around GWS and that the decision to pursue testing was an easy one. They appreciated the provision of educational material in GWS prior to pre‐test counseling
Luksic et al. (2020)	United States	To explore parental experiences of Clinical Exome Sequencing (CES) in a Latinx community, and to understand how their experiences are influenced by culture and language	Qualitative (interview)	38 parents	Parents wanted improved “contracting” or the dialogue exchange at the beginning of a session to establish a better rapport between the patient and provider
Lynch et al. (2021)	Australia	To understand parents' experiences of decision making for rGS within a rapid time frame (for critically unwell infants and children)	Qualitative (interview)	30 parents from 20 families	Parents wanted time to consider the implications of their rGS decision. There is also a need to evaluate informed consent in critical care contexts
Malek et al. (2019)	United States	To explore how parents of pediatric patients with cancer perceived the utility of clinical tumor and germline WES results	Quantitative (survey)	64 parents	Participants' roles as parents shaped their expectations about and experiences with ES. Themes of responsibility (participants' intentions to meet and set expectations they associate with parenthood) and culpability were often discussed
Malek et al. (2017)	United States	To understand parents' perspectives, perceptions, and experiences with genomic sequencing in the care of seriously ill children	Quantitative (survey)	122 parents	Even though parents were told as part of the consent process that participation in the study was highly unlikely to change their child's treatment plan, some still expressed hope that their child's WES results might provide clinical benefit. Some parents hoped that the results would offer exculpatory information to remove guilt
Mandrell et al. (2021)	United States	To describe parent perspectives of next generation sequencing (NGS) for their children with cancer, including perceived benefits, risks, hopes/expectations, and decision‐making process when consenting or not consenting to NGS and prior to result disclosure	Qualitative (interview)	42 parents	Parents endorsed more perceived benefits than risks, and the most frequent benefit was altruism. They also expressed the benefit of gaining knowledge, learning if cancer was hereditary, and the risk to other family members
Marshall et al. (2019)	Canada	To understand the personal utility of ES and the value of a diagnosis for parents of children with rare diseases	Quantitative (survey)	319 parents	Parents reported an increase in knowledge, changes in management, and access to services as the most valued attributes of ES
Martinussen et al. (2022)	Australia	To explore parental experiences of participation in the Victorian Undiagnosed Disease Program (UDP‐Vic) and the impact of receiving both diagnostic and undiagnostic sequencing results	Qualitative (interview)	21 parents	Parents discussed their experience of searching for a diagnosis and their need for “an answer.” Living without a diagnosis posed a challenge, so this was motivation for pursuing testing, a struggle stemming from uncertainty regarding the child's future and prognosis
Mccullough et al. (2016)	United States	To explore the expectations and attitudes of oncologists and parents of pediatric patients with cancer regarding genomic sequencing, specifically concerning the impact of ES on clinical decision‐making, clinical utility, and treatment expectations or outcomes	Qualitative (interview)	40 parents 16 oncologists	Parents stated knowing information was more important than not knowing the information, regardless of potential anxiety; contrary to being ethically disruptive, parents described their desire to take advantage of this new technology
Mollison et al. (2020)	United States	To determine how personal utility may be perceived by parents of pediatric patients who have undergone GWS, who had negative or uncertain results	Qualitative (interview) AND Clinical observation	31 parents	With a positive result, parents expressed relief that it was not something caused by them. This was followed by worry about future uncertainties related to the condition. With a negative result, it allowed parents to pause the diagnostic odyssey and turn attention to symptom management
Nguyen et al. (2025)	United States	To better understand the dynamics of communication in the diagnostic odyssey, we analyzed interview data from a prospective cohort study of the experiences of parents of undiagnosed children undergoing genomic sequencing for diagnosis of a presumed rare disease	Qualitative (interview)	32 parents	Parents discussed the need for trust and validation from providers. They specifically discussed challenges with coordination and continuity across providers, including a lack of recognition of the parent's knowledge of the child's symptoms and care needs
Odgis et al. (2025)	United States	To evaluate the impact of using video telehealth visits to return GS results to parents/legal guardians of children with suspected genetic conditions across two health systems in New York City	Quantitative (survey)		Parents discussed that telehealth was an effective modality for returning genomic results regardless of result type, though screen‐sharing visual aids did not significantly improve parental understanding or overall telehealth experience compared to controls
Outram et al. (2022)	United States	To explore parental expectations and value‐making processes with respect to pediatric clinical genomic sequencing for socially disadvantaged families	Qualitative (interview)	32 parents	Parents had hopes and expectations that came from their long diagnostic odysseys or from having a broad diagnosis. They hoped that sequencing would shed light on the future—their own, of other families, or of science
Pereira et al. (2021)	United States	To explore parents' and clinicians' attitudes towards perceptions of risks, benefits, and the utility of newborn GS vs. NBS prior to receiving study results	Quantitative (survey)	490 parents 144 clinicians	Only a minority of parents and clinicians believed that newborns should receive GS. They stated that informed consent should be required for GS and felt that more risks were associated with GS than NBS; Diagnosis and/or identification of risk was the main benefit noted by parents
Peter et al. (2022)	United Kingdom	To explore whether decisional regret and the psychological impact of receiving GS results differed between parents and patients, and between those who received a genetic diagnosis and those who did not	Mixed methods	296 parents completed the survey 38 interviews conducted	Parents found value in a label and said living without a clear diagnosis was difficult—especially from the perspective of explaining to schools, social services, and clinical care remained largely unchanged for the majority of cases (although it changed in a few cases); having a confirmation provided relief
Rosell et al. (2016)	United States	To explore the perceptions of parents on primary results of WES, specific perceptions explored were factors that would contribute to parental empowerment	Qualitative (interview)	19 parents	The majority of parents had high hopes for a diagnosis, although in some, it was tempered by prior experiences and not wanting to get their hopes up; a minority of parents had very low expectations. Parents hoped WES would provide targeted assistance in taking care of their child‐‐medical assistance or informing decisions about life‐planning for the child, as well as future reproductive decisions
Sanderson et al. (2019)	United Kingdom	To determine how healthcare professionals communicate with patients about consenting to GS	Clinical observation	35 patient/family member of the patient 10 healthcare professionals	No parental experiences were discussed as such. Much of the parental conversation was around consent scripts
Sapp et al. (2014)	United States	To characterize values and beliefs that shape parents' preferences for learning their children's sequencing results	Quantitative (survey)	25 parents	Parents described the diagnostic odyssey and desire to do the best to understand their child's condition. They noted that new information was not likely to cause significant additional stress, given what they had been through
Schwartz et al. (2022)	United States	To examine parental psychological health effects associated with their child's participation in genomic studies, particularly when parents meet the threshold for clinical concern for depression	Mixed methods	82 parents/families above threshold for clinical concerns 43 parents provided info for qualitative analysis	Parents discussed stress (or lack thereof) related to genomic RoR
Scollon et al. (2014)	United States	To better understand best‐practices for obtaining informed consent for ES in pediatric oncology settings	Review of EMR	100 parents	Parents didn't necessary mention specific experiences related to GS. Their conversations revolved around pediatric cancer care and associated supports
Scollon et al. (2019)	United States	To examine communication patterns and behaviors during the disclosure of ES results to parents of pediatric cancer patients	Mixed methods	40 parents	Parents expressed relief and Seeking Reassurance; Expression of concern and sense of responsibility (concerns about sharing with family members); seeking clarification of concepts and implications of results (carrier status was the most frequent concept that required clarification)
Sedig et al. (2022)	United States	To better understand adolescent and parent perspectives regarding motivations, attitudes, and knowledge related to paired tumor/germline sequencing	Quantitative (survey)	20 adolescents 77 parents	Parents and adolescents reported a good understanding of germline genetics and cancer risk, though parents had a better understanding. Both had similar motivations for participation—helping other children with cancer, helping to treat their child's/their specific cancer
Smith et al. (2019)	Canada	To understand parents' opinions regarding GWS and views towards secondary findings in a NICU setting, this study was done in comparison to another study (CAUSES study)	Quantitative (survey)	39 parents from 20 families	Parents identified multiple reasons they felt it was important to pursue GWS—namely, diagnosis and management; parents in the NICU setting seem to me more focused on immediacy as compared to parents in other settings, where parents are more focused on what can be done for their child over time
Stafford‐Smith et al. (2025)	United States	To describe the experiences of parents whose children were diagnosed with an ultra‐rare disease, and to explore empowerment in these parents related to the diagnosis	Qualitative (interview)	17 parents/19 families	Parents discussed that navigating their child's ultra‐rare diagnosis described emotional complexity and significant uncertainty, including concerns about prognosis, life expectancy, and emerging symptoms, with some noting their child was the oldest known case, leaving prognosis entirely uncharted
Stuckey et al. (2015)	United States	To develop a family genomic laboratory report designed to communicate genome sequencing results to parents whose children were participating in a WGS clinical research study	Qualitative (interview)	9 parents	Parents reported that more prognostic and condition‐specific information regarding next steps was needed; however, they were not able to articulate how this could be accomplished – Parents describe a search for valid information and resources regarding their child's condition – Parents believed that the Family Report would help facilitate communication with physicians and family members
Takatsuka et al. (2025)	Japan	To better understand parental needs and understand what factors contribute to environmental improvements in the lives of impacted children's families in Japan	Mixed methods	17 parents	Parents expressed relief and hope upon receiving a diagnosis, using it to plan for their child's future and support other families. An early diagnosis and clear return‐of‐results communication were important facilitators
Tolusso et al. (2017)	United States	To assess parents' perceived and actual understanding of clinical WES in their children to ultimately develop a validated tool to assess understanding of WES to be integrated into clinical care; the secondary purpose is to compare understanding of WES among parents of patients seen in genetics and non‐genetics specialty clinics	Quantitative (survey)	53 parents	The most common response provided by participants, regardless of their test status, was that it was important for providers to tell families that WES may not find a diagnosis for their child. Parents receiving positive results commented on the importance of discussing the implications of secondary findings, that WES could end a diagnostic odyssey, and that the results may change medical management
Townsend et al. (2012)	Canada	To understand attitudes about disclosure of secondary findings in WES from the perspective of genetics healthcare professionals, the general public, and parents whose children have experienced genetic testing	Qualitative (focus groups)	8 parents 10 healthcare professionals 10 members of the general public	All groups agreed that pre‐test discussions were crucial for patient‐informed decision‐making in order to avoid surprises of secondary findings. All groups thought to categorize secondary findings according to type, but none said that disclosure should be according to clinical relevance alone
Wainstein et al. (2022)	Canada	To investigate factors that influence decision‐making about rapid GS by parents of infants admitted to the NICU	Quantitative (survey)	16 parents	The article suggested that genetic counselors and other healthcare professionals might consider ensuring that discussions around rapid GS occur at a time which is most convenient for the parents, is scheduled ahead of time and does not overlap with other procedures occurring in the NICU to decrease or avoid any issues associated with anxiety
Werner‐Lin et al. (2019)	United States	To understand how families understand findings and adjust their perspectives on GWS	Qualitative (interview)	10 parents 10 adolescents	Parents and adolescents hoped for answers, and those who did not receive them expressed disappointment, although they did not hold providers responsible for this. Some hoped to learn detailed info about other family members, which was not necessarily the case
Williams et al. (2018)	United States	To test the effectiveness of an enhanced genomic report on patient‐centered outcome domains, including communication, engagement, and satisfaction	Mixed methods	52 participants	Most parents had no decisional regret regarding their child's participation in the WGS clinical research study. Parents who received a diagnosis found that the genetic report aided in communication with medical professionals. Parents repeatedly confirmed the challenges of interpreting and communicating genetic results
Wynn et al. (2018)	United States	To evaluate the parental experience, understanding, and psychological impact of ES by conducting a survey study of English‐speaking parents who had diagnostic ES	Quantitative (survey)	192 families	Parents wanted clarity about GWS in the informed consent process and effective communication of the clinician's interpretation of the child's results, along with an explanation of any differences in interpretation in the disclosure

### 3.3 Content analysis

Content analysis focused on the identification and classification of needs that were preceding, during, and following GWS testing for pediatric patients. We coded more quotations about needs preceding (39%) and following (57%) in contrast to during GWS (3.6%). Identified need categories centered on informational needs and emotional support, along with related sub‐categories. Table 3 provides information on categories and subcategories with supporting quotes. The information from additional articles found on December 3, 2025, did not change the observed themes.

**TABLE 3:** Categories and sub‐categories of needs with supporting quotes.

Need category	Example quote from text
Informational needs	
Tailoring communication and setting expectations	*Keeping pre‐test counseling brief and integrating this with a family systems approach can be very useful in a time‐limited situation and to enable decision‐making for parents who experience decisional conflict or moderate‐to‐severe anxiety*. (Hill et al., 2020) *Families being approached for genomic sequencing may benefit from clinicians highlighting the increased probability of a rare or novel gene finding when compared to traditional targeted genetic testing*. (Hendriks et al., 2005)
Information about logistics, prognosis, and next steps	*Parents across all interviews identified a desire to better understand their child's long‐term prognosis as a key motivator to pursue whole‐exome sequencing*. (Horton & Lucassen, 2019)
Future information changes	*We also found that parents…may want more information on certain topics, such as which genes had been tested and whether reanalysis would be available to them in the future*. (Gurasashvili et al., 2024; 11/8/24 11:00:00 AM)
Emotional impact and support	
Initial interactions with providers	*…we feel that many of the inequities revealed in our study could be addressed within the current model of genetic counseling, for example with improved ‘contracting,’ or the dialogue exchanged at the start of a genetic session that establishes the patient‐provider relationship*. (Lewis et al., 2020)
Support from providers and peer groups	*The importance of parent‐to‐parent support groups throughout the diagnostic journey was emphasized by parents in both cohorts, suggesting that peer connection may relieve social isolation for parents of children with rare disease*. (Hendriks et al., 2005)

#### 3.3.1 Informational needs

##### Providers need to tailor communication and set expectations appropriately

An overview of these articles indicated that parents expected clear communication from providers to help them understand the overall GWS process. In the pre‐test period, articles frequently emphasized parental reliance on providers to set and shape their expectations (Halley et al., 2022; Lee et al., 2022; Peter et al., 2022). This included the need for a clear and easy‐to‐understand informed consent process to set expectations for testing (Hill et al., 2020; Hitchcock et al., 2020; Mollison et al., 2020), and a desire for clarity and acknowledgment that GWS findings might not lead to different treatment or provide different prognostic information (Martinussen et al., 2022).

Tailoring pretest counseling conversations was discussed across different articles, based on both patient understanding and specific clinical scenarios. During the pretest period, specifically, needs varied even within similar contexts. For example, among studies in NICU or pediatric critical care settings, some parents preferred brief pretest conversations (Wainstein et al., 2022), while others expressed dissatisfaction when pretest information was limited (Brett et al., 2019).

##### Parents need ways to gain information about logistics, prognosis, and next steps

In the pre‐test period, some articles noted that a significant motivator for pursuing GWS was the desire to better understand prognosis (Alam et al., 2022). In the post‐test period, some articles found that parents also wanted information about logistics. Authors highlighted struggles with accessing medical services, medications, and dealing with insurance reimbursement issues (Halley et al., 2022). Some articles highlighted a need for an intermediary to guide them through the healthcare system (Krabbenborg, Vissers, et al., 2016), especially as the end of a diagnostic odyssey might lead to a new therapeutic journey requiring new care arrangements (Krabbenborg, Schieving, et al., 2016). Beyond clinical care, parents sought therapies and services that often required a genetic diagnosis to gain access (Childerhose et al., 2021). Some articles also reported a need for more information regarding secondary findings (Sanderson et al., 2019) as well as a clear understanding of how results might impact the management of their child's condition, including planned follow‐up, medical and otherwise (Krabbenborg, Schieving, et al., 2016; Liang et al., 2022; Stuckey et al., 2015).

##### Guidance about future information changes is needed

Articles discussed guidance about future information changes exclusively in the post‐test period. They discussed parents' desires to understand how variant interpretations might evolve and how recommendations could change over time, as well as seeking clarity on how the relationship between the clinical genetics team and parents might continue in order to address these desires (Hill et al., 2020). Some parents wanted explicit information on whom to contact and how to contact them (Krabbenborg, Vissers, et al., 2016), while others preferred periodic outreach from genetics professionals (Krabbenborg, Schieving, et al., 2016).

#### 3.3.2 Emotional impact and support

##### Initial interactions with providers are key

In the pre‐test period, initial interactions with the clinical team had an impact on parents' ability to process their feelings about the results in the post‐test period (Li et al., 2016). Furthermore, interactions with providers influenced parents' perceptions of accessibility and coordination of care; when providers maintained regular contact, parents felt their care was more accessible (Hitchcock et al., 2020). In both the interim period, while samples were being analyzed, and in the post‐test period, parents stated that improved accessibility of the clinic and associated providers improved rapport (Li et al., 2019).

##### Support from providers and peer groups is required

Some articles found a need for additional parental support in the pre‐test period related to their anxiety about genetic testing (Wainstein et al., 2022) and a desire to alleviate feelings of overwhelm (Luksic et al., 2020). In the post‐test period, articles mentioned a general need to feel emotionally supported by healthcare providers following the return of results (Krabbenborg, Schieving, et al., 2016; Liang et al., 2022). Other articles state that parents preferred that healthcare providers take a more active role in providing psychological support either by directing them towards resources or by making appropriate referrals (Malek et al., 2019; Werner‐Lin et al., 2018; Wynn et al., 2018). Some articles mentioned the desire for connection with support groups for children with similar diagnoses (Alam et al., 2022; Li et al., 2019). Many articles also indicated that parents wished genetics providers would be the conduit for access to such support groups (Krabbenborg, Schieving, et al., 2016; Krabbenborg, Vissers, et al., 2016; Martinussen et al., 2022; Rosell et al., 2016). Other articles highlighted the importance of acknowledging parental frustration and discussing the possibility of uncertain outcomes in the pretest period (Li et al., 2016). Discussions regarding return of results often co‐occurred with the concept of parental overwhelm and anxiety, typically in post‐test periods. Parents stated that receiving a diagnosis did not eliminate negative emotions such as anxiety (Peter et al., 2022).

Notably, very few articles discussed needs in the interim period between sample collection and the return of results.

## 4 DISCUSSION

In this scoping review, we examined 57 articles to determine the current state of knowledge about parental and caregiver needs while their children as patients underwent GWS. We found that their needs were interconnected and evolved across different stages of the GWS process. Two overarching categories of themes were identified: (1) Informational needs with the following sub‐categories: setting expectations and tailoring communication; information about prognosis, logistics, and next steps; and future information changes. (2) Emotional impact and support with the sub‐categories: initial interactions with providers and support from providers and peers.

This review highlights the fact that needs are varied across different stages but interconnected. In the pre‐test setting, parents sought information to understand the types of potential results and the logistical aspects of the GWS process. Prior research suggests that effective communication strategies and realistic pretest counseling are essential for helping families navigate the uncertainty and potential complexity of the GWS process. This emphasizes the importance of communication and suggests a need for more overarching support from healthcare systems themselves as parents navigate the healthcare landscape following genetic testing (Walton et al., 2020). In the post‐test setting, their needs shifted toward understanding the specific results they received, their medical implications, and guidance on how to use or interpret these results in the future. These needs are consistent with those of other articles where parents sought guidance about whether the diagnosis should lead to changes in the daily management of their child and the changing nature of GWS research (Krabbenborg, Schieving, et al., 2016). The review also suggests that the information provided and expectations set during the pre‐test period influenced how parents perceived and processed information in the post‐test period. This finding underscores the importance of tailoring communication to align with parents' understanding of genetic information and emotional state in the pre‐test setting, as well as setting appropriate expectations, to support better comprehension and decision‐making in the post‐test period. The extension of benefit from pre‐test counseling into the post‐test setting, specific to GWS, regardless of diagnostic yield, has been seen previously in the literature (Manickam et al., 2021).

In post‐test periods, our findings suggest that parents also need appropriate expectation setting, now focused on both the immediate and long‐term future. Parents expressed the need to understand how test results may impact medical care and access to non‐medical services. They also sought clarity on how GWS results and associated recommendations may evolve with future reanalysis or new data that could impact clinical guidance. Other articles examining post‐diagnostic care in rare disease contexts also indicate that genetic conditions can pose a challenge to traditional care management models. This is because patients often need follow‐up care and support from different categories of health professionals, from several different medical specialties, as well as social work providers, all of which require a level of coordination challenging to organize in most health care systems (Ferrara et al., 2017).

Our findings highlight that initial interactions with healthcare providers were also crucial for establishing rapport and offering emotional support to parents. Parents experienced anxiety and feelings of overwhelm throughout the GWS process. In the pre‐test period, this anxiety stemmed from the uncertainty of potential results, while in the post‐test period, feelings of overwhelm were linked to the complexity and volume of information provided. This suggests that anxiety persists across both stages of the GWS process and underscores the need for providers to deliver information empathetically to help manage parental anxiety and overwhelm. Furthermore, while parents expressed a desire for information, overburdening parents with information appeared to exacerbate feelings of overwhelm, indicating that a careful balance in communication is essential. Other similar articles report that balancing the amount of information provided does not result in lower parental understanding, and framing information in a way that allows patients to leverage their understanding can allow them to gain agency in their medical care (Joseph et al., 2019).

This review also emphasizes the importance of tailoring communication to match parents' and caregivers' level of understanding and emotional state. This highlights the interconnected nature of informational and emotional needs, as meeting informational needs can directly influence emotional well‐being. Parents especially valued and emphasized feeling cared for, having continuous relationships with clinicians, receiving empathetic communication, being kept informed while awaiting genetic test results, and being connected with informational and psychosocial resources following results (Crellin et al., 2023). This particular sentiment is echoed in other literature, where framing information in a way that enabled patients to leverage their understanding and gain agency over their medical decision‐making also had psychosocial benefits (Joseph et al., 2019). Effectively addressing patients' informational needs helps them feel supported throughout the process, and establishing a good rapport early on further enhances their sense of emotional support.

Parents sought support not only from healthcare providers but also from peer support groups, suggesting that they benefit from diverse perspectives beyond just the medical viewpoint. This appears to indicate that parents gained support from a sense of solidarity through interactions with peers who experienced similar medical challenges. This is seen in other clinical genetics literature where parents recruited through peer support groups have emphasized the value of social and informational resources provided by these groups (Nelson Goff et al., 2013; Riggan et al., 2021). Notably, our review indicates that parents rely on healthcare providers to facilitate connections to resources outside the clinical setting, such as peer support networks. Thus, healthcare providers remain integral, either as direct providers of emotional support or as conduits to other supportive resources for parents.

Additionally, there are very limited studies that are examining needs during the interim period between sample collection and return of GWS results. As such, there is a relative dearth of literature addressing how to support families during the interim period between sample collection and return of results. While GWS increasingly has short turnaround times, which may reduce the duration of this interim period, we argue that it remains clinically significant for several reasons. First, even with rapid turnaround times, the interim period represents a high‐anxiety waiting phase for families. Second, and perhaps more importantly, a proportion of families undergoing GWS do not receive a definitive answer, creating an expectation of hope for future reanalysis or reinterpretation. This observation prompts a re‐evaluation of what “post‐test” truly means in the context of GWS. Unlike traditional genetic testing that may be conceptualized as “one and done,” GWS often initiates an ongoing process of periodic reanalysis and reinterpretation as genetic knowledge evolves. Therefore, the interim period may not be a discrete phase between pre‐ and post‐test counseling, but rather an extended state that many families experience as they await future answers. Recognizing this, we identified the need to better understand and address family support during these interim periods, whether measured in weeks during initial testing or years while awaiting reanalysis. While this temporal lens for examining needs (pre‐test, interim, and post‐test) is established in genetic counseling practice, its explicit application to needs assessment research may offer a useful framework for identifying gaps in family support, particularly during often‐overlooked interim periods.

Lastly, although several articles noted that parents required support at specific points during the GWS process, the specific nature of these needs was not articulated. This highlights a significant knowledge gap in the literature regarding parental needs in GWS. Addressing these identified gaps presents an opportunity for future research to further explore and define these specific support needs. Future studies may also benefit from directly engaging with parents of children who have undergone GWS to gain insights into their experiences and assess how their needs align with or diverge from those outlined in this review.

### 4.1 Limitations

The findings of this scoping review should be considered in light of several limitations. Included articles were primarily from high‐income countries and were all in English, which may limit the generalizability of these findings to diverse cultural, socioeconomic, and geographic contexts. Articles with negative or inconclusive results, or those conducted in lower‐resourced settings, may be underrepresented in the literature due to publication bias. This has the potential to skew the findings toward more well‐supported themes, potentially overlooking important challenges faced in less studied or less well‐resourced environments. Additionally, the inclusion criteria for this review were limited to peer‐reviewed articles, which may miss important insights from gray literature. Despite these limitations, this scoping review highlights what is known in the literature and sheds light on what could be further explored with regard to parental needs in GWS.

## 5 CONCLUSION

In conclusion, this study identified informational and emotional support needs that spanned across different points of the GWS process in various clinical settings. The examination of these needs through contiguous points in the GWS process sheds light on how informational and support needs evolve over the course of the process and how they are interconnected across time and through healthcare systems. By understanding these needs and how they arise, providers who are involved in different parts of the GWS process can better support families through the complex and often overwhelming process of pediatric GWS, ostensibly improving the implementation of GWS into pediatric care.

## AUTHOR CONTRIBUTIONS

**Priyanka Murali:** Conceptualization; data curation; formal analysis; investigation; methodology; project administration; supervision; writing – original draft; writing – review and editing. **Joon‐Ho Yu:** Conceptualization; methodology; resources; supervision; writing – review and editing.

## CONFLICT OF INTEREST STATEMENT

The authors state no conflicts of interest.

## ETHICS STATEMENT

Human studies and informed consent: This scoping review was in accordance with ethical research principles, utilizing only publicly available published data from peer‐reviewed sources. As such, no informed consent or ethical approval was required, and the study did not involve direct interaction with human participants or the collection of personally identifiable data.

Animal studies: Not applicable.

## Supporting information


Appendix S1–S4

## Data Availability

Data available on request.
