# Supplementary material for: Parent/caregiver needs during pediatric genome‐wide sequencing: A scoping literature review
Source: J Genet Couns. 2026 Apr 30;35:e70209. doi: 10.1002/jgc4.70209 (PMC13131311; doi:10.1002/jgc4.70209)
Supplement: Supplementary file 1 — Appendix S1–S4 [file JGC4-35-0-s001.docx]

**Supplemental information –**

Appendix S1.

**Scoping Review Protocol**
**Design Plan**

Study type: Other

Blinding: No blinding is involved in this study.

Study design: We will conduct a scoping review to understand the state of the literature as it pertains to parent and caregiver needs as they go through the process of pediatric genome-wide sequencing. We will then attempt to categorize these needs temporally and in different clinical contexts.

Randomization: None

**Sampling Plan**

Data collection procedures:

We will conduct a scoping review and extract data from the studies about parent and caregivers needs as they go through the process of GWS. We plan to conduct searches in the following databases: Pubmed, CINAHL, PsycInfo, Web of Science, and Embase. Three researchers will parse through the data consistent with scoping review. We will use the extracted data to develop a broader clinical framework for understanding parental needs in pediatric GWS.

Sample size: We will be searching five databases (PubMed, Embase, Web of Science, Cinahl, and PsycInfo). We do not know how many articles will be returned until we run the searches.

Sample size rationale**:** We are searching all databases that may have relevant articles and do not have significant overlap.

Stopping rule**:** We will see how many search results we have and determine how many of those articles are relevant to our question of interest.

**Variables**

Manipulated variables

Measured variables: These will include our search strings for each database.

**Analysis Plan**

Statistical models **–** n/a

Transformations – N/a

Inference criteria **–** N/a

Data exclusion: Studies where parent/caregiver data could not be isolated from other participant groups; Studies where GWS results were not returned to parents/caregivers; Non-English language articles

Missing data**:** n/a

Appendix S2.

Summary of inclusion and exclusion criteria

| Framework Element | Inclusion Criteria | Exclusion Criteria |
| --- | --- | --- |
| Population | Parents and caregivers of pediatric patients (aged 0-18 years) who had undergone GWS; Studies with multiple participant groups if parent/caregiver data could be isolated | Studies where parent/caregiver data could not be isolated from other participant groups |
| Concept | Parent/caregiver experiences, expectations, and perceptions related to GWS; Parent/caregiver needs (preceding, during, and following GWS); Stakeholder interactions; GWS results must have been returned to parents/caregivers | Studies where GWS results were not returned to parents/caregivers |
| Context | Any clinical subspecialty; Any geographic location; No restrictions on setting | None |
| Types of Sources | English-language articles; Qualitative, quantitative, and mixed methods studies; GWS defined as whole exome or whole genome sequencing | Non-English language articles |

Appendix S3.

**Complete search strings by database**

PubMed

(Infant[Mesh] OR Child[Mesh] OR Adolescent[Mesh] OR Pediatrics[Mesh] OR Infan*[tiab] OR newborn*[tiab] OR "new-born*"[tiab] OR prematur*[tiab] OR preterm*[tiab] OR perinat*[tiab] OR neonat*[tiab] OR baby*[tiab] OR babies[tiab] OR toddler*[tiab] OR minors[tiab] OR minors*[tiab] OR boy[tiab] OR boys[tiab] OR boyhood[tiab] OR girl*[tiab] OR kid[tiab] OR kids[tiab] OR child*[tiab] OR schoolchild*[tiab] OR "school-age*"[tiab] OR adolescen*[tiab] OR juvenil*[tiab] OR youth*[tiab] OR teen*[tiab] OR "under-age*"[tiab] OR pubescen*[tiab] OR pediatric*[tiab] OR paediatric*[tiab] OR peadiatric*[tiab])

**AND**

("Whole Genome Sequencing"[MeSH Terms] OR "Exome sequencing"[MeSH Terms] OR "genome sequenc*"  OR “genomic sequenc*” OR "exome sequenc*" OR “genetic sequenc*" OR “gene panel sequenc*” OR “genetic screening”)

**AND**

("parent attitudes"[tiab:~2] OR "parent's attitudes"[tiab:~2] OR "parents' attitudes"[tiab:~2] OR "parental attitudes" OR

"parent perspective"[tiab:~2] OR "parent's perspective"[tiab:~2] OR "parents' perspective"[tiab:~2] OR "parental perspective"[tiab:~2] OR

"parent perspectives"[tiab:~2] OR "parent's perspectives"[tiab:~2] OR "parents' perspectives"[tiab:~2] OR "parental perspectives"[tiab:~2] OR

"parent needs"[tiab:~2] OR "parent's needs"[tiab:~2] OR "parents' needs"[tiab:~2] OR "parental needs"[tiab:~2] OR

"parent knowledge"[tiab:~2] OR "parent's knowledge"[tiab:~2] OR "parents' knowledge"[tiab:~2] OR "parental knowledge"[tiab:~2] OR

"parent experiences"[tiab:~2] OR "parent's experiences"[tiab:~2] OR "parents' experiences"[tiab:~2] OR "parental experiences"[tiab:~2] OR "parent choice"[tiab:~2] OR "parent's choice"[tiab:~2] OR "parents' choice"[tiab:~2] OR "parental choice"[tiab:~2] OR "parent choices"[tiab:~2] OR "parent's choices"[tiab:~2] OR "parents' choices"[tiab:~2] OR "parental choices"[tiab:~2] OR "parent opinions "[tiab:~2] OR "parent's opinions "[tiab:~2] OR "parents' opinions "[tiab:~2] OR "parental opinions "[tiab:~2] OR "parent preference"[tiab:~2] OR "parent's preference"[tiab:~2] OR "parents' preference"[tiab:~2] OR "parental preference"[tiab:~2] OR "parent preferences"[tiab:~2] OR "parent's preferences"[tiab:~2] OR "parents' preferences"[tiab:~2] OR "parental preferences"[tiab:~2] OR "parent concerns "[tiab:~2] OR "parent's concerns"[tiab:~2] OR "parents' concern "[tiab:~2] OR "parental concerns"[tiab:~2] OR

“parent belief"[tiab:~2] OR "parent's belief"[tiab:~2] OR "parents' belief"[tiab:~2] OR "parental belief"[tiab:~2] OR

“parent beliefs"[tiab:~2] OR "parent's beliefs"[tiab:~2] OR "parents' beliefs"[tiab:~2] OR "parental beliefs"[tiab:~2] OR

"parent desire"[tiab:~2] OR "parent's desire"[tiab:~2] OR "parents' desire"[tiab:~2] OR "parental desire"[tiab:~2] OR

"parent desires"[tiab:~2] OR "parent's desires"[tiab:~2] OR "parents' desires"[tiab:~2] OR "parental desires"[tiab:~2] OR

"parent perception"[tiab:~2] OR "parent's perception"[tiab:~2] OR "parents' perception"[tiab:~2] OR "parental perception"[tiab:~2] OR

"parent perceptions"[tiab:~2] OR "parent's perceptions"[tiab:~2] OR "parents' perceptions"[tiab:~2] OR "parental perceptions"[tiab:~2] OR

(Parents[Mesh] AND (“Health knowledge, attitudes, practice”[Mesh] OR Attitude[Mesh])))

Web of Science

(Infan* OR newborn* OR "new-born*" OR prematur* OR preterm* OR perinat* OR neonat* OR baby* OR babies OR toddler* OR minors OR minors* OR boy OR boys OR boyhood OR girl* OR kid OR kids OR child* OR schoolchild* OR "school-age*" OR adolescen* OR juvenil* OR youth* OR teen* OR "under-age*" OR pubescen* OR pediatric* OR paediatric* OR peadiatric*)

**AND**

(“genome sequenc*” OR “genomic sequenc*” OR "exome sequenc*" OR “genetic sequenc*" OR “gene panel sequenc*” OR “genetic screening”)

**AND**

(parent* NEAR/1 (attitudes OR perspective* OR needs OR knowledge OR experiences OR choice* OR opinions OR preference* OR belief* OR desire* OR perception*))

CINAHL (EBSCO)

**(**(MH "Child+") OR (MH "Infant+") OR (MH "Adolescence+") OR (MH "Pediatrics+") OR

TI(Infan* OR newborn* OR "new-born*" OR prematur* OR preterm* OR perinat* OR neonat* OR baby* OR babies OR toddler* OR minors OR minors* OR boy OR boys OR boyhood OR girl* OR kid OR kids OR child* OR schoolchild* OR "school-age*" OR adolescen* OR juvenil* OR youth* OR teen* OR "under-age*" OR pubescen* OR pediatric* OR paediatric* OR peadiatric*) OR

AB(Infan* OR newborn* OR "new-born*" OR prematur* OR preterm* OR perinat* OR neonat* OR baby* OR babies OR toddler* OR minors OR minors* OR boy OR boys OR boyhood OR girl* OR kid OR kids OR child* OR schoolchild* OR "school-age*" OR adolescen* OR juvenil* OR youth* OR teen* OR "under-age*" OR pubescen* OR pediatric* OR paediatric* OR peadiatric*)**)**

 AND

**(**(MH "Genetic Screening+") OR

TI(“genome sequenc*” OR “genomic sequenc*” OR "exome sequenc*" OR “genetic sequenc*" OR “gene panel sequenc*” OR “genetic screening”)

OR

AB(“genome sequenc*” OR “genomic sequenc*” OR "exome sequenc*" OR “genetic sequenc*" OR “gene panel sequenc*” OR “genetic screening”)**)**

AND

**(**

(((MH "Attitude+") OR (MH "Health Knowledge")) AND (MH "Parents+")) OR

TI(parent* N1 (attitudes OR perspective* OR needs OR knowledge OR experiences OR choice* OR opinions OR preference* OR belief* OR desire* OR perception*))

**OR**

AB(parent* N1 (attitudes OR perspective* OR needs OR knowledge OR experiences OR choice* OR opinions OR preference* OR belief* OR desire* OR perception*))

**)**

Psycinfo (EBSCO)

**(**

AG(Childhood OR Adolescence)

OR

MA(“Infant” OR “Child” OR “Adolescent” OR “Pediatrics”)

 OR

KW(Infan* OR newborn* OR "new-born*" OR prematur* OR preterm* OR perinat* OR neonat* OR baby* OR babies OR toddler* OR minors OR minors* OR boy OR boys OR boyhood OR girl* OR kid OR kids OR child* OR schoolchild* OR "school-age*" OR adolescen* OR juvenil* OR youth* OR teen* OR "under-age*" OR pubescen* OR pediatric* OR paediatric* OR peadiatric*)

**OR**

TI(Infan* OR newborn* OR "new-born*" OR prematur* OR preterm* OR perinat* OR neonat* OR baby* OR babies OR toddler* OR minors OR minors* OR boy OR boys OR boyhood OR girl* OR kid OR kids OR child* OR schoolchild* OR "school-age*" OR adolescen* OR juvenil* OR youth* OR teen* OR "under-age*" OR pubescen* OR pediatric* OR paediatric* OR peadiatric*)

**OR**

AB(Infan* OR newborn* OR "new-born*" OR prematur* OR preterm* OR perinat* OR neonat* OR baby* OR babies OR toddler* OR minors OR minors* OR boy OR boys OR boyhood OR girl* OR kid OR kids OR child* OR schoolchild* OR "school-age*" OR adolescen* OR juvenil* OR youth* OR teen* OR "under-age*" OR pubescen* OR pediatric* OR paediatric* OR peadiatric*)

**)**

**AND**

**(**

DE ("Genomic Sequencing")

OR

MA ("Whole Genome Sequencing" OR "Exome sequencing")

OR

KW(“genome sequenc*” OR “genomic sequenc*” OR "exome sequenc*" OR “genetic sequenc*" OR “gene panel sequenc*” OR “genetic screening”)

OR

TI(“genome sequenc*” OR “genomic sequenc*” OR "exome sequenc*" OR “genetic sequenc*" OR “gene panel sequenc*” OR “genetic screening”)

**OR**

AB(“genome sequenc*” OR “genomic sequenc*” OR "exome sequenc*" OR “genetic sequenc*" OR “gene panel sequenc*” OR “genetic screening”)**)**

**AND**

**(**

((DE "Parents" OR DE "Adoptive Parents" OR DE "Expectant Parents" OR DE "Fathers" OR DE "Foster Parents" OR DE "Homosexual Parents" OR DE "Mothers" OR DE "Parental Characteristics" OR DE "Single Parents" OR DE "Stepparents" OR DE "Surrogate Parents (Humans)") AND (DE "Parental Attitudes" OR DE "Parental Expectations" OR DE "Health Attitudes" OR DE "Attitudes" OR DE "Health Behavior" OR DE "Health Knowledge"))

OR

MA ((Parents AND (“Health knowledge, attitudes, practice” OR Attitude))

OR

KW(parent* N1 (attitudes OR perspective* OR needs OR knowledge OR experiences OR choice* OR opinions OR preference* OR belief* OR desire* OR perception*))

**OR**

TI(parent* N1 (attitudes OR perspective* OR needs OR knowledge OR experiences OR choice* OR opinions OR preference* OR belief* OR desire* OR perception*))

**OR**

AB(parent* N1 (attitudes OR perspective* OR needs OR knowledge OR experiences OR choice* OR opinions OR preference* OR belief* OR desire* OR perception*))

**)**

**Embase (Elsevier)**

**(**'adolescent'/exp OR 'child'/exp OR 'infant'/exp OR 'pediatrics'/exp

OR

(Infan* OR newborn* OR "new-born*" OR prematur* OR preterm* OR perinat* OR neonat* OR baby* OR babies OR toddler* OR minors OR minors* OR boy OR boys OR boyhood OR girl* OR kid OR kids OR child* OR schoolchild* OR "school-age*" OR adolescen* OR juvenil* OR youth* OR teen* OR "under-age*" OR pubescen* OR pediatric* OR paediatric* OR peadiatric*):TI,AB,KW**)**

**AND**

('whole exome sequencing'/exp OR 'whole genome sequencing'/de

OR

(“genome sequenc*” OR “genomic sequenc*” OR "exome sequenc*" OR “genetic sequenc*" OR “gene panel sequenc*” OR “genetic screening”):TI,AB,KW**)**

**AND**

**((**'parent'/exp AND ('attitude to health'/exp OR 'attitude'/exp))

OR
(parent* NEAR/2 (attitudes OR perspective* OR needs OR knowledge OR experiences OR choice* OR opinions OR preference* OR belief* OR desire* OR perception*)):TI,AB,KW**)**

Appendix S4.

PRISMA checklist

| **Section and Topic** | **Item #** | **Checklist item** | **Location where item is reported** |
| --- | --- | --- | --- |
| **TITLE** | | |  |
| Title | 1 | Parent/caregiver needs during pediatric genome-wide sequencing: a scoping literature review | P1 |
| **ABSTRACT** | | |  |
| Abstract | 2 | See the PRISMA 2020 for Abstracts checklist. | P2L2 – 22 |
| **INTRODUCTION** | | |  |
| Rationale | 3 | Describe the rationale for the review in the context of existing knowledge. | P3L25 – P4L7 |
| Objectives | 4 | Provide an explicit statement of the objective(s) or question(s) the review addresses. | P4L8 – 13 P4L25 – 26 |
| **METHODS** | | |  |
| Eligibility criteria | 5 | Specify the inclusion and exclusion criteria for the review and how studies were grouped for the syntheses. | P5L18 – 22 |
| Information sources | 6 | Specify all databases, registers, websites, organisations, reference lists and other sources searched or consulted to identify studies. Specify the date when each source was last searched or consulted. | P5L6 |
| Search strategy | 7 | Present the full search strategies for all databases, registers and websites, including any filters and limits used. | P5L4 – 16 |
| Selection process | 8 | Specify the methods used to decide whether a study met the inclusion criteria of the review, including how many reviewers screened each record and each report retrieved, whether they worked independently, and if applicable, details of automation tools used in the process. | P6L8 – 16 |
| Data collection process | 9 | Specify the methods used to collect data from reports, including how many reviewers collected data from each report, whether they worked independently, any processes for obtaining or confirming data from study investigators, and if applicable, details of automation tools used in the process. | P6L8 – 16 |
| Data items | 10a | List and define all outcomes for which data were sought. Specify whether all results that were compatible with each outcome domain in each study were sought (e.g. for all measures, time points, analyses), and if not, the methods used to decide which results to collect. | P6L17 – 7L8 |
|  | 10b | List and define all other variables for which data were sought (e.g. participant and intervention characteristics, funding sources). Describe any assumptions made about any missing or unclear information. | P6L17 – 7L8 |
| Study risk of bias assessment | 11 | Specify the methods used to assess risk of bias in the included studies, including details of the tool(s) used, how many reviewers assessed each study and whether they worked independently, and if applicable, details of automation tools used in the process. | P7L6 – 8 |
| Effect measures | 12 | Specify for each outcome the effect measure(s) (e.g. risk ratio, mean difference) used in the synthesis or presentation of results. | P7L6 – 8 |
| Synthesis methods | 13a | Describe the processes used to decide which studies were eligible for each synthesis (e.g. tabulating the study intervention characteristics and comparing against the planned groups for each synthesis (item #5)). | P7L6 – 8 |
|  | 13b | Describe any methods required to prepare the data for presentation or synthesis, such as handling of missing summary statistics, or data conversions. | P7L6 – 8 |
|  | 13c | Describe any methods used to tabulate or visually display results of individual studies and syntheses. | P7L6 – 8 |
|  | 13d | Describe any methods used to synthesize results and provide a rationale for the choice(s). If meta-analysis was performed, describe the model(s), method(s) to identify the presence and extent of statistical heterogeneity, and software package(s) used. | P7L6 – 8 |
|  | 13e | Describe any methods used to explore possible causes of heterogeneity among study results (e.g. subgroup analysis, meta-regression). | P7L6 – 8 |
|  | 13f | Describe any sensitivity analyses conducted to assess robustness of the synthesized results. | P7L6 – 8 |
| Reporting bias assessment | 14 | Describe any methods used to assess risk of bias due to missing results in a synthesis (arising from reporting biases). | n/a |
| Certainty assessment | 15 | Describe any methods used to assess certainty (or confidence) in the body of evidence for an outcome. | n/a |
| **RESULTS** | | |  |
| Study selection | 16a | Describe the results of the search and selection process, from the number of records identified in the search to the number of studies included in the review, ideally using a flow diagram. | P7L24 – P8L3; Flow diagram in supplemental materials |
|  | 16b | Cite studies that might appear to meet the inclusion criteria, but which were excluded, and explain why they were excluded. | n/a |
| Study characteristics | 17 | Cite each included study and present its characteristics. | Supplemental materials |
| Risk of bias in studies | 18 | Present assessments of risk of bias for each included study. | n/a |
| Results of individual studies | 19 | For all outcomes, present, for each study: (a) summary statistics for each group (where appropriate) and (b) an effect estimate and its precision (e.g. confidence/credible interval), ideally using structured tables or plots. | n/a |
| Results of syntheses | 20a | For each synthesis, briefly summarise the characteristics and risk of bias among contributing studies. | n/a |
|  | 20b | Present results of all statistical syntheses conducted. If meta-analysis was done, present for each the summary estimate and its precision (e.g. confidence/credible interval) and measures of statistical heterogeneity. If comparing groups, describe the direction of the effect. | n/a |
|  | 20c | Present results of all investigations of possible causes of heterogeneity among study results. | n/a |
|  | 20d | Present results of all sensitivity analyses conducted to assess the robustness of the synthesized results. | n/a |
| Reporting biases | 21 | Present assessments of risk of bias due to missing results (arising from reporting biases) for each synthesis assessed. | n/a |
| Certainty of evidence | 22 | Present assessments of certainty (or confidence) in the body of evidence for each outcome assessed. | n/a |
| **DISCUSSION** | | |  |
| Discussion | 23a | Provide a general interpretation of the results in the context of other evidence. | P11L1 – P14L9 |
|  | 23b | Discuss any limitations of the evidence included in the review. | P14L10 – 20 |
|  | 23c | Discuss any limitations of the review processes used. | P14L10 – 20 |
|  | 23d | Discuss implications of the results for practice, policy, and future research. | P11L1 – P14L9 |
| **OTHER INFORMATION** | | |  |
| Registration and protocol | 24a | Provide registration information for the review, including register name and registration number, or state that the review was not registered. | P4L27 – P5L2 |
|  | 24b | Indicate where the review protocol can be accessed, or state that a protocol was not prepared. | P4L27 – P5L2 |
|  | 24c | Describe and explain any amendments to information provided at registration or in the protocol. | n/a |
| Support | 25 | Describe sources of financial or non-financial support for the review, and the role of the funders or sponsors in the review. | P1L10 |
| Competing interests | 26 | Declare any competing interests of review authors. | P1L11 |
| Availability of data, code and other materials | 27 | Report which of the following are publicly available and where they can be found: template data collection forms; data extracted from included studies; data used for all analyses; analytic code; any other materials used in the review. | P1L9 |
